# Paediatric cardiac arrest prognostication in the context of a HEMS service

**DOI:** 10.1186/s13049-024-01174-5

**Published:** 2024-01-22

**Authors:** Benjamin Stretch

**Affiliations:** Queen Mary University, London, UK

Many thanks to Fuchs et al. for publishing their retrospective study looking at neurological outcome following out of hospital paediatric cardiac arrest, which showed a high rate of positive neurological outcome (19%) [1].

Firstly, when looking at prognostic factors, I believe traumatic and non-traumatic cardiac arrest should be analysed separately. This is emphasised by the significant difference in survival shown in this study (OR 11.07), a different treatment algorithm recommended by the European Resuscitation Council and a focus on different reversible causes [2]. For example, there is some debate over the role of chest compressions in traumatic cardiac arrest– bystander CPR and adrenaline may have limited prognostic value in the context of severe hypovolaemia, tension pneumothorax or cardiac tamponade. As a result, we may find that the positive neurological predictors in trauma may differ to those identified overall in this study.

This study is a cohort of HEMS patients resulting in a higher number of traumatic cardiac arrests in rural locations which is not representative of all cause paediatric arrests. As expected, a key predictor of neurological outcome seems to be time. Both direct measures (response time / time to BLS > 2 min) and indirect measures (ongoing CPR at HEMS arrival / adrenaline doses) of low-flow and no-flow time seem to correlate closely with neurological outcome. This study raises the question of the exact role of HEMS in the management of cardiac arrest, where early basic intervention rather than complex advanced treatments deliver the best prognostic value. HEMS teams can deliver advanced interventions which may be of additional value optimising physiology after return of spontaneous circulation. Different interventions are required in traumatic cardiac arrest which may be beyond the scope of paramedic teams. In some systems, there may also be a time advantage– both in delivering care to the patient (for example in rural areas) and delivering the patient to definitive care.

It is important to emphasize that early bystander CPR was an important prognostic factor, with 33% of patients in this study not receiving this. This is an excellent reminder of the importance of education, particularly in the context of paediatrics. An example of this includes the Resuscitation Council United Kingdom’s ‘Aaron’s Heart’ - a free educational book on paediatric resuscitation, produced in response to a survey showing that only 15% parents would recognise if their child was in cardiac arrest [3]. 

## Data Availability

No data presented.
